# The course of subjective and objective chemosensory dysfunction in hospitalized patients with COVID-19: a 6-month follow-up

**DOI:** 10.1007/s00405-021-06796-4

**Published:** 2021-04-10

**Authors:** Mattis Bertlich, Clemens Stihl, Enzo Lüsebrink, Johannes C. Hellmuth, Clemens Scherer, Saskia Freytag, Jennifer Lee Spiegel, Ivelina Stoycheva, Martin Canis, Bernhard G. Weiss, Friedrich Ihler, Frank Haubner

**Affiliations:** 1 Department of Otorhinolaryngology, University Hospital, Head and Neck Surgery, Ludwig-Maximilians University of Munich, Marchioninistr. 15, 81377 Munich, Germany; 2 Medizinische Klinik und Poliklinik I, University Hospital, Ludwig-Maximilians-University of Munich, Marchioninistr. 15, 81377 Munich, Germany; 3 Medizinische Klinik und Poliklinik III University Hospital, Ludwig-Maximilians-University of Munich, Marchioninistr. 15, 81377 Munich, Germany; 4 Epigenetics and Genomics Division, Harry Perkins Institute of Medical Research, Nedlands, WA Australia

**Keywords:** SARS-CoV-2, Coronavirus, Hyposmia, Anosmia, COVID-19, Chemosensory dysfunction, Smell training

## Abstract

**Purpose:**

It has been established that the infection with SARS-CoV-2 may cause an impairment of chemosensory function. However, there is little data on the long-term effects of SARS-CoV-2 infection on chemosensory function.

**Methods:**

Twenty three SARS-CoV-2-positive patients diagnosed in spring 2020 with subjective hyposmia (out of 57 positive patients, 40.3%) were compared to SARS-CoV-2-positive patients without hyposmia (*n* = 19) and SARS-CoV-2-negative patients (*n* = 14). Chemosensory function was assessed by the Brief Smell Identification Test (BSIT), Taste Strips (TS), Visual Analogue Scales (VAS), and the SNOT-22. The initial cohort with hyposmia were also examined at 8 weeks and 6 months after initial examination.

**Results:**

There were no differences between the SARS-CoV-2-positive cohort without hyposmia and negative controls in terms of BSIT (8.5 ± 2.6 vs. 10.2 ± 1.8), TS (3.4 ± 0.6 vs. 3.9 ± 0.3) or VAS (2.1 ± 1.3 vs. 1.1 ± 0.5); yet the SNOT-22 was significantly elevated (27.7 ± 11.2 vs. 16.4 ± 10.8). The SARS-CoV-2-positive group with hyposmia performed significantly poorer in BSIT (4.0 ± 1.7 vs. 8.5 ± 2.6/10.2 ± 1.8), TS (2.6 ± 1.3 vs. 3.4 ± 0.6/3.9 ± 0.3), and VAS (7.9 ± 2.2 vs. 2.1 ± 1.3/1.1 ± 0.5) compared to both control groups. At week 8 and month 6 control, six and five patients, respectively, still suffered from subjectively and objectively impaired chemosensory function. The other patients had recovered in both respects.

**Conclusion:**

SARS-CoV-2 patients with subjectively impaired chemosensory function regularly perform poorly in objective measurements. About 70% of patients suffering from olfactory dysfunction in SARS-CoV-2 quickly recover—the rest still suffers from considerable impairment 6 months after infection.

## Introduction

The coronavirus SARS-CoV-2 first rose to global attention in December 2019, when it was regularly found to cause severe pneumonia [1]. The symptom complex was later named COVID-19. Due to its high contagiosity it quickly spread to Europe and the Americas and caused an unprecedented social and economic disruption with its (relatively) high lethality. The WHO considered COVID-19 to be a “a public health emergency of international concern”, on the 30th of January and even updated it to be a “pandemic” as of 11th of March, 2020.

Initial research on the virus focused strongly on its origin, genetic properties, individual risk factors, and potential treatments. This was ratiocinative, given the pressing matters at hand. However, as time passed and coping with SARS-CoV-2 gradually became routine, starting in April reports arose that an impaired sense of smell and/or taste was a common symptom of infection with SARS-CoV-2 [2–4]. Subsequent quantitative data on the matter showed that a considerable amount of patients that tested positive for SARS-CoV-2 actually suffered from impaired sense of smell [5].

However, olfactory dysfunction has only been investigated in a minor extent—compared to other aspects of COVID-19. As it has been suggested that a great majority of patients suffering from chemosensory dysfunction eventually resolve symptoms within a short period of time, [6, 7] there is—to this day—little quantitative data available on long-term effects of SARS-CoV-2 infection on smell and taste function. This is particularly important as especially long-term chemosensory dysfunction is associated with a number of severe psychosocial disorders as decreased sexual attraction [8] and increased risk for depression [9, 10].

As there is little data available on the long-term effects of infection with SARS-CoV-2 on individual perception, we conducted a retrospective chart review of the long-term course of chemosensory dysfunction after SARS-CoV-2 infection.

## Materials and methods

The study at hand was registered with the ethics committee of the Ludwig-Maximilians University (Munich, Federal Republic of Germany) under the file number 20-253. Patients in this study that had tested positive for SARS-CoV-2 were also included in the prospective CORKUM (CORona virus disease at the Klinikum der Universtität München) study under the file no. 20-245 The study was conducted in compliance with the Declaration of Helsinki from 2000.

**Fig. 1:**
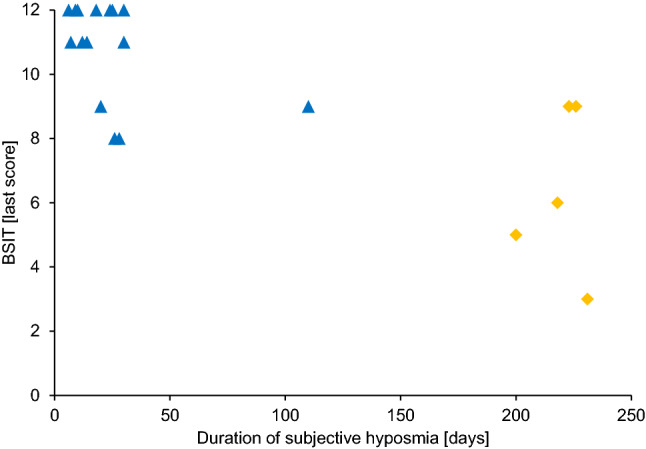
Duration of subjectively impaired chemosensory function and the last available objective measurements of smell (BSIT). Blue = Chemosensory impairment had resolved at the last examination, yellow = chemosensory impairment was still present at the last examination

All patients that had been registered with the ICD-10 code U07.1 (COVID-19) and had tested positive for SARS-CoV-2 by polymerase chain reaction in the spring of 2020 were screened for this study. Patients that had been tested positive for SARS-CoV-2 during spring 2020 were divided into two groups—patients that stated to suffer from subjective impairment of chemosensory function (smell and/or taste) and those that did not. Patients were asked regularly whether they had been experiencing impaired sense of smell and/or taste as part of the routine work-up during their inpatient stay. A third group of patients that were scheduled for ear surgery and had no prior history of conditions of the nose or paranasal sinuses was used as a control, as these patients routinely underwent SARS-CoV-2 testing pre-operatively (Fig. 1).

(Electronic) patient files were then used to obtain information on the patients individual and COVID-19 specific characteristics. Individual characteristics included age, gender, general diseases, medication that was taken on a regular base. COVID-19 characteristics included the way of infection as well as the date, the time of onset of symptoms and symptoms other than hyposmia that have been reported to occur in COVID-19, such as odynophagia, fever, tiredness, dry cough, sneezing, myalgia, nasal obstruction, nausea, diarrhea and hemoptysis.

Chemosensory dysfunction was measured by the “Brief Smell Identification Test” (BSIT). The BSIT is an abbreviated version of the University of Pennsylvania Smell Identification Test (UPSIT), a supra-threshold odor identification test that has been established as a reliable measurement of smell function [11]. The BSIT (purchased from Sense Trading, Groningen, The Netherlands) contains 12 odors and was administered as recommended by the provider: Patients were handed the booklet containing the 12 odors. Upon scratching each of the 12 contact fields with a pencil, patients were asked to pick one out of four answers as to what smell they have been exposed to. It has been shown that BSIT values strongly correlate with the UPSIT [12]. As recommended in recent literature, values equal to or smaller than nine out of twelve were considered to be a indicative of impaired smell (hyposmia), [12] values equal to or smaller than four were considered to be indicative of anosmia. For the taste examination, taste strips that have been used and validated extensively were used [13]. For each taste, only the highest concentrations were used so as to determine whether the patient was able to taste each quality or not. The SNOT-22 was filled out by the patients digitally via a QR-code as provided by the software ENT-Statistics (Innoforce Est, Rugell, Principality of Liechtenstein). If the patient had no handheld electronic device capable of reading a QR-code, the SNOT-22 was completed verbally with an ENT-specialist. Moreover, patients were asked to grade the amount of chemosensory dysfunction on a visual anaolgue scale ranging from 1 (no impairment at all) to 10 (highest possible impairment).

Patients that had stated to suffer from impaired chemosensory function were subsequently examined at scheduled follow-up visits at sister departments or were asked to participate in follow-up examinations either in an outpatient setting or by mail. Follow-up examinations were performed 8 weeks after initial examination and after 6 months. During follow-ups, patients were asked if they still suffered from subjective impairment of chemosensory function, to grade the impairment on a visual analogue scale and were asked to complete the B-SIT and taste strip examinations as well as the SNOT-22.

Statistical analysis was carried out using RStudio for Mac (Version 1.2.1335, RStudio Inc., Boston, MA, United States of America). A *p* value of smaller than 0.05 was considered to be significant.

## Results

Initially, 57 patients that tested positive during April 2020 for SARS-CoV-2 by polymerase chain reaction and had been hospitalized were screened for this study. Average age was 64.9 ± 14.7 years. 42 were male and 15 female. Out of these 23 patients reported to experience impaired chemosensory function (40.3%). Those patients made up the positive cohort that suffered subjective hyposmia.

Out of these 23 patients, 17 (77.3%) were male and six female. Average age was 59.0 ± 16.6 years. Average score on the SNOT-22 was 24.2 ± 9.8. Subjective chemosensory impairment (VAS) was graded on average with 7.9 ± 2.2. The B-SIT showed an average of 4.0 ± 1.7 points (out of 12 points). 16 patients suffered from anosmia and six patients suffered from hyposmia. In terms of taste strips, patients scored on average 2.6 ± 1.3 points (out of 4). 10 patients showed normogeusia, 11 showed hypogeusia and one patient showed ageusia as per the previously defined criteria. As of the last follow-up, there was no mortality amongst this group of patients (Table 1).

**Table 1:** Comparison of characteristics between baseline groups

	SARS-CoV-2-positive with Hyposmia (*n* = 23)	SARS-CoV-2-positive without Hyposmia (*n* = 19)	SARS-CoV-2-negative (*n* = 14)
Age (years)	59.0 ± 16.6	68.0 ± 17.3	61.7 ± 18.0
Gender (male)	17 (77.3%)	15 (78.9%)	10 (71.4%)
SNOT-22	24.2 ± 9.8	27.7 ± 11.2	16.4 ± 10.8
VAS	7.9 ± 2.2	2.1 ± 1.3	1.1 ± 0.5
B-SIT	4.0 ± 1.7	8.5 ± 2.6	10.2 ± 1.8
Normosmia	0 (0.0%)	9 (47.3%)	10 (71.4%)
Hyposmia	6 (27.3%)	8 (42.1%)	4 (28.6%)
Anosmia	16 (72.7%)	2 (10.5%)	0 (0.0%)
Taste strips	2.6 ± 1.3	3.4 ± 0.6	3.9 ± 0.3
Normogeusia	10 (45.5%)	16 (84.2%)	14 (100.0%)
Hypogeusia	11 (50.0%)	3 (15.8%)	0 (0.0%)
Ageusia	1 (4.5%)	0 (0.0%)	0 (0.0%)
Mortality	0 (0.0%)	4 (21.1%)	0 (0.0%)

Statistics (*p* values)			
	SARS + H + vs. SARS + H −	SARS + H + vs. SARS −	SARS + H – vs. SARS −
SNOT-22	0.383^#^	**< 0.001**^#^	**0.008**^#^
VAS	**< 0.001**^#^	**< 0.001**^#^	**< 0.001**^#^
BSIT (absolute values)	**0.002**^#^	**< 0.001**^#^	0.5936^#^
BSIT (interpretation)	**< 0.001**^**†**^	**< 0.001**^**†**^	0.4537^†^
Taste strips (absolute values)	0.067^#^	**0.014**^#^	0.2444^#^
Taste strips (interpretation)	**0.013**^**†**^	**< 0.001**^†^	0.2443^†^

Values are given as mean ± standard deviation. Bold indicates *p* < 0.05, # = Fisher’s exact test, ^†^ = Wilcoxon signed rank test

Compared to this, controls that were tested positive for SARS-CoV-2 but that did not suffer from subjective impairment of chemosensory function consisted of 19 patients. These were recruited after the initial cohort had been recruited. Out of these, 15 (78.9%) were male and four (21.1%) were female. Average age was 68.0 ± 17.3 years. On average, patients scored 27.7 ± 11.2 points on the SNOT-22 and reported chemosensory function to be 2.1 ± 1.1 on a visual analogue scale. There was no statistically significant difference between these patients and the positive patients with Hyposmia, but a significant difference between those patients and the negative controls. Patients scored on average 8.5 (out of 12) ± 2.6 on the BSIT. Out of those, nine (47.3%) patients showed normosmia, eight patients (42.1%) showed hyposmia and two patients (10.5%) showed anosmia. This was significantly different compared to patients positive for SARS-CoV-2 with hyposmia but not compared to negative controls. In terms of taste, patients scored 3.4 ± 0.6, resulting in 16 patients with normogeusia (84.2%), three patients with hypogeusia (15.8%) and no patients with ageusia. This was only significantly different from SARS-CoV-2-positive patients with hyposmia in respect to the interpretation of the taste strips. Four of the 19 patients had died in the course of the disease (Table 1).

The control group that had tested negative for SARS-CoV-2 was on average 61.7 ± 18.0-years-old. Out of those patients, ten (71.4%) were male. Participants scored on the SNOT 10 on average 16.4 ± 10.8 points. In terms of the individual analogue scale, patients scored 1.1 ± 0.5 points. Patients scored on average 10.2 ± 1.8 odors on the BSIT, resulting in ten (71.4%) patients with normosmia, four (28.6%) with hyposmia and no patients with anosmia. In terms of objective taste, all patients showed normogeusia (100.0%).

In brief, both groups that had tested positive for SARS-CoV-2 were comparable in terms of comorbidities. In terms of symptoms, a sore throat, coughing and cephalgia was more common in the group that suffered from subjectively impaired chemosensory function (Table 2).

**Table 2:** Comparison of symptoms and comorbidities between SARS-CoV-2 positive groups with and without Hyposmia

	SARS-CoV-2-positive with Hyposmia (*n* = 23)	SARS-CoV-2-positive without Hyposmia (*n* = 19)	Odds ratio	*p*
Comorbidities				
Immunosuppression	3 (13.0%)	4 (21.1%)	0.570 [0.039–5.074]	0.682
Pulmonary diseases	2 (8.7%)	3 (15.8%)	0.516 [0.132–2.748]	0.516
Arterial hypertension	13 (56.5%)	9 (47.3%)	1.432 [0.362–5.807]	0.757
Malignoma	1 (4.3%)	5 (26.3%)	0.133 [0.002–1.370]	0.075
Pollinosis	1 (4.3%)	1 (5.3%)	0.822 [0.009–67.719]	1.000
History of nicotin abuse	3 (13.0%)	6 (31.6%)	0.334 [0.046–1.901]	0.257
Symptoms				
Sore throat	6 (26.1%)	0 (0.0%)	∞ [1.104–∞]	**0.024**
Fever	20 (87.0%)	15 (78.9%)	1.753 [0.254–13.820]	0.682
Tiredness	18 (78.3%)	10 (52.6%)	3.145 [0.711–15.631]	0.107
Cough	20 (87.0%)	9 (47.4%)	7.020 [1.377–49.542]	**0.008**
Myalgia	10 (43.5%)	3 (15.8%)	3.966 [0.795–27.147]	0.093
Cephalgia	10 (43.5%)	1 (5.3%)	13.082 [1.528–631.146]	**0.006**
Diarrhea	4 (17.4%)	0 (0.0%)	∞ [0.573–∞]	0.193
Emesis	2 (8.7%)	0 (0.0%)	∞ [0.156–∞]	0.493

Bold writing indicates *p* < 0.05 by Fisher’s exact test

At the 8-week follow-up, twenty patients reported back. Out of these, six (30.0%) still claimed to suffer from impaired chemosensory function and 14 reported that impairment had resolved. The patients in which subjective hyposmia had resolved suffered from hyposmia for an average of 18.5 ± 8.4 days (7–30 days). Average scores on the SNOT-22 were 17.6 ± 7.6 points, on the VAS were 3.8 ± 2.6 points, on the B-SIT 9.0 ± 3.1 points and 3.6 ± 0.8 points for the taste strips. Subsequently, 11 patients showed normosmia and eight showed hyposmia. 18 patients showed normogeusia and two hypogeusie. The respective values for the individual groups that showed persistent impairment of chemosensory function and those that did not can be found in Table 3.

**Table 3:** Characteristics of patients with subjective hyposmia over time; H + = subjective hyposmia subjective present at examination, H −  = no subjective hyposmia present anymore at examination

	Baseline April 2020 (*n* = 23)	Follow-up 8 weeks			Follow-up 6 months		
		∑ (*n* = 20)	H + (*n* = 6)	H − (*n* = 14)	∑ (*n* = 17)	H + (*n* = 5)	H − (*n* = 12)
SNOT-22	24.2 ± 9.8	17.6 ± 7.6	22.8 ± 6.6	15.3 ± 6.8	20.3 ± 7.7	25.4 ± 6.2	18.2 ± 7.3
VAS	7.9 ± 2.2	3.8 ± 2.6	7.2 ± 1.3	2.4 ± 1.3	3.8 ± 2.7	7.6 ± 1.4	2.3 ± 1.1
B-SIT	4.0 ± 1.7	9.0 ± 3.1	5.0 ± 1.4	10.7 ± 1.7	9.4 ± 2.6	6.4 ± 2.3	10.6 ± 1.1
Normosmia	0 (0.0%)	11 (55.0%)	0 (0.0%)	11 (78.6%)	10 (58.8%)	0 (0.0%)	8 (66.7%)
Hyposmia	6 (27.3%)	8 (40.0%)	5 (83.3%)	3 (21.4%)	6 (35.3%)	4 (80.0%)	4 (33.3%)
Anosmia	16 (72.7%)	1 (5.0%)	1 (16.7%)	0 (0.0%)	1 (5.9%)	1 (20.0%)	0 (0.0%)
Taste strips	2.6 ± 1.3	3.6 ± 0.8	3.7 ± 0.5	3.6 ± 0.9	3.7 ± 0.5	3.2 ± 0.4	3.9 ± 0.3
Normogeusia	10 (45.5%)	18 (90.0%)	6 (100.0%)	12 (85.7%)	17 (100.0%)	4 (100.0%)	12 (100.0%)
Hypogeusia	11 (50.0%)	2 (10.0%)	0 (0.0%)	2 (14.3%)	0 (0.0%)	0 (0.0%)	0 (0.0%)
Ageusia	1 (4.5%)	0 (0.0%)	0 (0.0%)	0 (0.0%)	0 (0.0%)	0 (0.0%)	0 (0.0%)

Values are given as mean ± standard deviation

At the 6-month follow-up, 17 patients reported back. Out of those, five (29.4%) patients stated to still suffer from subjective impairment of chemosensory function. Patients scored on average 20.3 ± 7.7 points on the SNOT-22. Impairment of chemosensory function was graded on average with 3.8 ± 2.7 points on the VAS. Patients scored on average 9.4 ± 2.6 out of twelve odors in the B-SIT. 10 patients showed normosmia, six patients still showed hyposmia and one patient showed persistent anosmia. In the taste strip examination, average score was 3.7 ± 0.5. All patients showed normogeusia. Individual values for patients that still claim to experience impaired chemosensory function can be found in Table 3.

A survival analysis with a cox proportional hazards models showed no influence of either the SNOT-22 (*p* = 0.7), the VAS (*p* = 0.3) or the B-SIT (*p* = 0.8) on the persistence of subjective chemosensory dysfunction.

## Discussion

It has been established—albeit relatively late during this pandemic—that infection with SARS-CoV-2 may cause chemosensory dysfunction [5, 6]. There has been a large meta-analysis by Hannum and colleagues that reported the prevalence of chemosensory dysfunction in SARS-CoV-2 infection to be as high as 77% (95% confidence interval 61.4–89.2%) [14]. Taking into account all patients that had tested positive for SARS-CoV-2 in the study at hand, we found that 78% of these showed impaired chemosensory function, which is in line with the aforementioned study and speaks for the validity of the cohort at hand.

When it comes to impaired function of chemosensors, most scientific publications that report quantitative objective data focus on olfaction. Publications that focus on subjective measures, as is very common during this pandemic, cannot—by nature—clearly distinguish between olfaction and taste. There are few studies that actually tried to measure taste by psychophysical examinations [15, 16]. Both studies found an impairment of gustatory function during acute infection, that, albeit not statistically significantly, recovered after infection. It has been suggested that this impairment may actually be caused by a missing central nervous interaction of taste and smell [17]. In respect to taste the dataset at hand is in line with the literature available on this topic.

Moreover, there is—to this date—very little data available on the course of chemosensory dysfunction in SARS-CoV-2 infection. It has been reported that approximately two thirds of cases with objective hyposmia resolve within 4 weeks after infection [6, 18]. The only study that has examined the medium-term outcomes of chemosensory function after SARS-CoV-2 infection was conducted by Niklassen et al. [15] who reported that 27% of patients showed persisting hyposmia more than 4 weeks after the infection. These findings are in line with the results reported in the study at hand. D’Ascanio and colleagues reported similarly that the majority of patients with subjective impairment of chemosensory function recover within 30 days, which again is in line with the results presented in the study at hand [18]. Interestingly, the patients that had not recovered at the 8-week follow-up only rarely recovered until the 6-months follow-up. Bearing this in mind, we believe that there are two distinct courses of hyposmia in SARS-CoV-2 Infection: The majority (about ¾) of patients that suffer from subjectively impaired chemosensory function recover within the course of 4 weeks spontaneously and need no subsequent treatment. However, if chemosensory impairment lasts longer than this, a longer lasting impairment seems probable. Unfortunately, the dataset at hand suggests there is no way of determining who is to suffer from longer lasting impairment upon the onset of symptoms. If olfactory dysfunction persists, olfactory training has been recommended as the treatment of choice, potentially with intranasal sodium citrate or vitamin A [19]. Particularly olfactory training has been shown to be very effective in post-inflammatory hyposmia, [20, 21] as is the case in hyposmia after SARS-CoV-2 infection.

It has long been established in scientific literature that in respect to chemosensory function, subjective measures are not reliable [22, 23]. However, the dataset at hand provided evidence that in (and even after) SARS-CoV-2 Infection, there may be some merit to the subjective assessment of chemosensory function: There is a considerable proportion of patients—both in the control groups and in the follow-ups—that claim to not suffer from impaired chemosensory function but still performed poorly in psychophysical examinations. On the other hand, every patient that claimed to actually suffer from impaired chemosensory function performed poorly in in the psychophysical examinations. Subsequently, we believe that while a subjectively unimpaired chemosensory function is not indicative of no infection with SARS-CoV-2, subjectively impaired chemosensory function may very well be indicative of an underlying infection.

The SNOT-22 seems—at least in the study at hand—to be able to differentiate between patients that have previously tested positive for SARS-CoV-2. However, it does not differentiate between patients that are suffering from subjectively impaired chemosensory function and those that do not. This is probably due to the fact that several items in the SNOT-22 are questions about general wellbeing. Fittingly, a study from the United Arab Emirates found that patients with more severe courses of COVID-19 regularly score higher on these items [24]. Bearing in mind that the collective at hand consists of patients that were hospitalized, these patients regularly scoring higher in the SNOT-22 seems logical. Consequently, the validity of the SNOT-22 seems to be impaired in patients with concomitant COVID-19 disease.

## Conclusion

The majority of patients that suffer from subjectively impaired chemosensory function during active COVID-19 infection actually show impaired sense of smell and/or taste in psychophysical testing. Patients that do not state to suffer from impaired chemosensory function do not necessarily show normal results in psychophysical testing. Moreover, the majority of patients that do suffer from subjectively impaired chemosensory function recover within 4 weeks after the onset of symptoms. However, those patients that do not recover within those 4 weeks tend to suffer from these symptoms even 6 months after the infection and exhibit fittingly impaired psychophysical test results. These patients should be informed about olfactory training and potential additional treatments such as sodium citrate and intranasal vitamin a.

## Data Availability

The original, anonymous dataset is available upon request from the corresponding author.

## Abbreviations

BSIT: Brief smell identification test
COVID-19: Coronavirus disease 2019
SNOT-22: Sino-nasal outcome test (22 Items)
TS: Taste strips
VAS: Visual Analogue Scale

## Declarations

### Dataset

The authors of this manuscript declare that the data at hand have not been published, submitted or used in any other manuscript elsewhere.
